# Webcam-based eye-tracking to measure visual expertise of medical students during online histology training

**DOI:** 10.3205/zma001642

**Published:** 2023-09-15

**Authors:** Dogus Darici, Carsten Reissner, Markus Missler

**Affiliations:** 1 Westfälische-Wilhelms-University, Institute of Anatomy and Neurobiology, Münster, Germany

**Keywords:** digital histology, eye-tracking study, histology training, online education, visual expertise, visual expertise development, webcam eye-tracking, webcam eye-tracking methodology

## Abstract

**Objectives:**

Visual expertise is essential for image-based tasks that rely on visual cues, such as in radiology or histology. Studies suggest that eye movements are related to visual expertise and can be measured by near-infrared eye-tracking. With the popularity of device-embedded webcam eye-tracking technology, cost-effective use in educational contexts has recently become amenable. This study investigated the feasibility of such methodology in a curricular online-only histology course during the 2021 summer term.

**Methods:**

At two timepoints (t1 and t2), third-semester medical students were asked to diagnose a series of histological slides while their eye movements were recorded. Students’ eye metrics, performance and behavioral measures were analyzed using variance analyses and multiple regression models.

**Results:**

First, webcam-eye tracking provided eye movement data with satisfactory quality (*mean accuracy*=115.7 px±31.1). Second, the eye movement metrics reflected the students’ proficiency in finding relevant image sections (*fixation count on relevant areas*=6.96±1.56 vs. irrelevant areas=4.50±1.25). Third, students’ eye movement metrics successfully predicted their performance (R^2^_adj_=0.39, p<0.001).

**Conclusion:**

This study supports the use of webcam-eye-tracking expanding the range of educational tools available in the (digital) classroom. As the students’ interest in using the webcam eye-tracking was high, possible areas of implementation will be discussed.

## Zusammenfassung

**Ziel:**

Visuelle Expertise spielt eine wichtige Rolle bei der Prozessierung von Bildern, wie sie häufig in der Radiologie oder Histologie vorkommen. Studien zeigen, dass sich mit zunehmender visueller Expertise die Augenbewegungen der Untersuchenden verändern. Die Entwicklungen im Webcam-Eye-Tracking ermöglichen seit einiger Zeit den kostengünstigen und flächendeckenden Einsatz dieser Methode. Die vorliegende Studie untersuchte die Qualität dieser Technologie in einem Histologie-Kurs im Sommersemester 2021.

**Methoden:**

An zwei Zeitpunkten wurden Medizinstudierende im dritten Semester gebeten, eine Reihe histologischer Präparate zu beurteilen. Währenddessen wurden ihre Augenbewegungen aufgezeichnet und gemeinsam mit der Performanz, sowie behavioralen Parameter mittels Varianzanalysen und multipler Regressionsmodelle analysiert.

**Ergebnisse:**

Die Ergebnisse zeigten, dass Webcam-Eye-Tracking qualitativ hochwertige Daten liefern kann (mittlere Genauigkeit=115.7 px±31.1). Außerdem spiegelten Augenbewegungen die Fähigkeit der Teilnehmenden wider, relevante Bildbereiche zu finden (Fixationsanzahl relevanter Bereiche=6.96±1.56 vs. irrelevanter Bereiche = 4.50±1.25). Schließlich konnten die Augenbewegungen der Teilnehmenden deren Leistung vorhersagen (R^2^_adj_=0.39, p<0.001).

**Diskussion:**

Diese Studie unterstützt den Einsatz von Webcam-Eye-Tracking Technologie zur Erfassung von visueller Expertise. Da auch die Akzeptanz hoch war, werden mögliche Implementierungsoptionen diskutiert.

## Introduction

To date, several studies have investigated the eye movement patterns of expert and novice diagnosticians [4], [6], [23], [26], [27], [29], [30], [33], [35], [39], [49]. Differences in eye movement have been associated with levels of medical expertise and related to diagnostic accuracy [12], [39]. For example, expert diagnosticians in histopathology direct their focus to relevant image sections more frequently, longer, and faster than novices [4], [35]. These differences in visual behavior have been identified in a variety of domains, ranging from chess [18] to pilot training [62], as well as medical applications such as radiography [14], [33], [36], electrocardiogram interpretation [53], diagnostic decision-making [52] and histopathology [4], [6], [26], [35]. Most of these studies have been carried out using modern near-infrared eye-tracking devices, which precisely record eye movements and make them available for an in-depth examination.

Although this methodology seems to have the potential in measuring visual expertise, its widespread use in medical education has been sparse due to several implementation hurdles [16], [32]. First, modern eye trackers are expensive, i. e., often exceeding $10,000 per device. Second, the application of such technology is very personnel intensive, as it is necessary to have a trained scientist present during both the calibration and the study procedure. Third, the analysis of the data requires special training since large amounts of data are collected and more advanced statistical methods are applied. As a result, such trackers have mostly been used in specialized laboratories in a controlled environment. To overcome these limitations, computer scientists have recently sought to develop new solutions. The most promising approach has been conducted by Papoutsaki and colleagues. They developed an open-source JavaScript code that captures eye movements using web cameras built into everyday devices such as laptops, tablets, and cell phones [44], [45]. Since then, this technology has found its way into user-friendly online platforms with graphical user interfaces. In addition, execution and calibration were automated. This approach combines numerous advantages at the same time; i.e., it is less expensive, widely available, and easy to use. It enables participants to engage in online studies in a more naturalistic home environment at convenient times, hence increasing the likelihood of successful data collection. However, especially eye-tracking based investigations are vulnerable when unsupervised, because variations in participants’ behavior greatly affects the data quality [25].

The present study attempts to address these limitations by examining the use of webcam eye trackers in a curricular histology course. During the online only course – as necessitated by the COVID-19 pandemic – a large cohort of medical students was assessed. Conceptually, this study refers to visual expertise, a construct that has a long history in the eye-tracking literature [24], [46] and has been studied in the field of histopathology training [4], [26]. Visual expertise provides a suitable interpretive framework for the collected data, allows the metrics to be placed in a theoretical context, and turns abstract concepts into measurable observations.

### Visual expertise in histopathology

Visual expertise can be defined as the complex interplay between perceptual and cognitive processes that evolves by training and leads to higher accuracy in image search, recognition, and decision-making [15]. Originating in chess research, it is now assumed to be one of the main learning goals in courses that rely on visual cueing, such as histopathology, gross anatomy, and radiology [47], [59]. Capturing eye movements is a promising method for measuring visual expertise, as it is considered to occur involuntarily and allows for the in-depth evaluation of visual pattern recognition competencies [32]. Thus, the eye-tracking methodology enables us to gain a better understanding of the mechanisms underlying visual expertise development. This approach is supported by the eye-mind hypothesis, which postulates a direct connection between eye movements and “what the mind is engaged with” [1], [28].

Existing literature on visual expertise in histopathology studied the eye movement behavior of expert pathologists [6], [26]. Usually, these measurements were compared with those of novices in an expert-novice paradigm. It is assumed that novices develop visual expertise when their visual behaviors reach the level of those of experts. These processes are assumed to occur unconsciously, thereby reflecting procedural pattern-recognition competencies and tacit knowledge [8], [31], [32], [34].

### Theoretical concepts of visual expertise

The most influential and empirically supported theories that try to explain visual expertise are holistic processing theory and the information reduction model [19], [36], [51]. Holistic processing postulates that visual experts show a more integrated image perception, which allows them to rapidly focus on diagnostically relevant areas of interest (*dAOI*) on the slide. The time it takes to direct the gaze on dAOIs (*time to first fixate dAOIs*) has thus been associated with a higher level of visual expertise [51]. This visual behavior is neurobiologically facilitated through an increased parafoveal vision [51], which implies that expert diagnosticians capture a wider field of view of information when viewing images. This enables them to discover important areas earlier and move their gaze on them more quickly. It is therefore to be expected that the time it takes to solve a task (*view time*) will likewise shorten with an increase in expertise [4], [6]. Another prominent theory is the information-reduction model, which closely relates to the idea of selective processing [19]. Briefly, this theory assumes that expert diagnosticians – in order to save mental resources – neglect diagnostically irrelevant information, while shifting their focus to dAOIs. In contrast, novice learners fail to detect dAOIs but move their gaze toward visually salient yet diagnostically redundant areas (*vAOI*) [6]. Thus, according to the information-reduction model, successful visual expertise development can be operationalized by more frequent and longer *fixations of dAOIs*, as well as less frequent and shorter *fixations of vAOIs*.

Many findings related to the differences between experts and novices can usefully be explained by an overlap of the abovementioned theories, in which eye-tracking enables the measurability of important propositions of these theories.

### Research questions

#### Research question 1: How accurately can webcam eye-tracking detect eye movements? 

A suitable methodology would translate into good *accuracy, precision* measurements, high *data integrity* (=little data loss), and acceptable *sampling rates* [24], [25]. 

#### Research question 2: How valid is webcam eye-tracking in regard to capturing changes in visual expertise? 

We expected that students would develop visual expertise in the histology course. According to holistic processing theory and information reduction model, this development would show up in increased *test scores* and reduced view times but also in changes in eye movements, such as a reduced* time to first fixation of dAOI*, as well as higher *fixation counts on dAOI*. We hypothesized an opposite trend with visually salient but task-redundant regions of *vAOIs*.

#### Research question 3: How reliably can webcam eye-tracking distinguish between low and high performances? 

As eye movements are a predictor of visual expertise [4], interindividual differences should be predictable based on eye movement. Thus, statistical models should predict the students’ test scores based on their eye movement data.

## Methods

This study was conducted at the Westfälische Wilhelms-University in Münster during the summer term of 2021. At two particular timepoints, one third-semester preclinical cohort was evaluated longitudinally alongside an online-only histology course. More details can be retrieved from the supplementary files (see attachment 1 ).

### Participating students

The first measurement (t1) was conducted after 10 three-hour sessions. Here, *N*=51 students (age *mean* 21.56±2.21 years; 35 females) were included for data analysis. The second measurement (t2) was conducted after 20 three-hour sessions immediately before a written examination, as an improvement in students’ visual expertise can be expected at such a point. N=77 students (age *mean* 21.97±2.25 years; 59 females) were included in t2. Informed consent was received from all students. This study was carried out in accordance with the Declaration of Helsinki. The study protocol was reviewed by the ethics committee (“Ethik-Kommission der Ärztekammer Westfalen-Lippe und der Westfälischen Wilhelms-University”) and deemed not to require formal medical ethics approval.

### Study procedure of the webcam eye-tracking study

**Figure 1:**
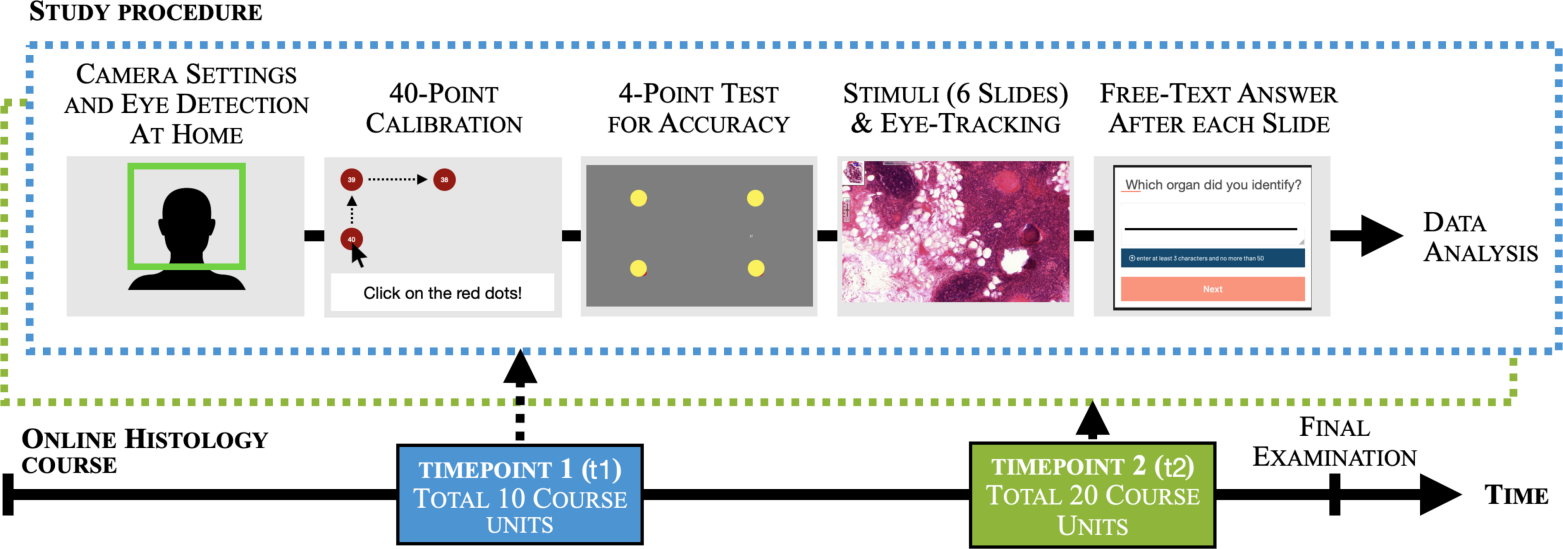
The study procedure is shown schematically. At two timepoints during an online-only histology course, one cohort of third semester medical students passed both a 9-point eye-tracking calibration and a 4-point test for eye-tracking accuracy. Afterward, they were instructed to identify 6 histological slides each (*slide identification task*). The test score, view time and several eye movements were recorded. Data were preprocessed and analyzed.

**Figure 2:**
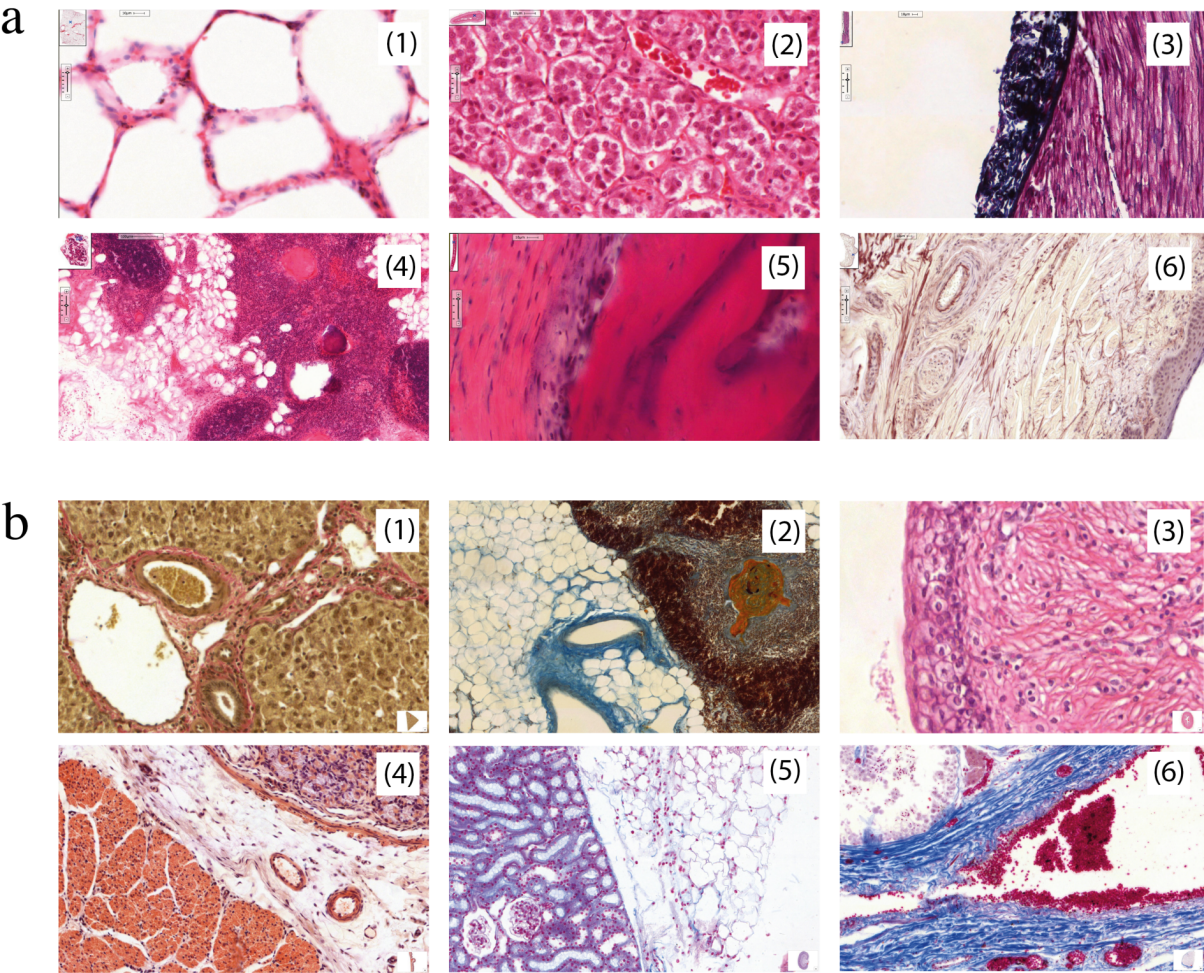
Histological slides used in this study (name+staining). (a) Slides shown at timepoint one (t1): 1) Alveoli in Hematoxylin Eosin; 2) Adrenal glands in H.E.; 3) Peripheral nerve in Azan; 4) Thymus in H.E.; 5) Bone in H.E.; 6) Skin – Finger in Elastica. (b) Slides shown at timepoint two (t2): 1) Liver in van Gieson; 2) Thymus in Azan; 3) Ureter in H. E.; 4) Stomach in Van Gieson; 5) Kidney in Azan; 6) Testis in Azan.

The study design corresponds to a single-group pre-post intervention design with a measurement interval of 7 weeks (=10 course sessions) (see attachment 1 ). Due to the COVID-19 pandemic, the complete semester cohort was obliged to participate in the synchronous online-only course. A run-through pilot study was performed with two participants to optimize the eye-tracking environment. Here, the main focus was to adjust the duration of the presentation time and to assess the behavior during the study. The actual study participants were recruited during the online course and received a hyperlink that led to the online study. They could perform the study at home anytime during a period of one week around the two timepoints. After starting the study, the students passed a 40-point eye-tracking calibration and a 4-point test for accuracy (see figure 1 (Fig. 1) and figure 2 a (Fig. 2)). The participants next looked at six histology slides for a maximum of 15 seconds each. After each slide, the participants were prompted to identify the organ on the slide. Meanwhile, the test score, view time, and eye movements were recorded.

### Online eye-tracking with web cameras

An open-source JavaScript code (WebGazer) was used to record the binocular gaze position [45]. The study took approximately 10-15 minutes and ran entirely on a web browser in full view mode; no additional software was needed. No personal image data were transmitted during the session, as the JavaScript code runs locally on the participant’s computer. The output provided the respective binocular X and Y coordinates with a timestamp, and subject IDs. We offered e-mail support for students with technical problems (*n*=1 at t_2_).

### Description of the stimuli and instruction 

Six different histological slides were shown at each of the two timepoints, and care was taken to ensure that the level of difficulty was approximately the same (see figure 2 (Fig. 2)). Different slides were used at both time points to prevent the recognition of the slides based on nonspecific patterns (e.g., staining). These slides were instructed with a slide identification task: “identify the following organ”. From our own experience in oral examinations, we think that the rapid identification of histological slides is a selective task for novice students. Slides were presented in the same order and for a maximum duration of 15 seconds. The view time was deliberately kept short, both to increase the overall difficulty and to capture early search behavior and rapid pattern recognition competencies. Scrolling or zooming was disabled to reduce the complexity for the students and enhance comparability at the expense of authenticity. Students who finished the task in less than 15 seconds could skip to the questioning to prevent idle eye movements. Returning to an image was not possible.

### Description of the test score

To reduce the probability of incidental answers [21] and to make sure students did not simply guess the right multiple-choice answer by chance, the participants were asked free text questions (e.g., “Which organ did you identify?”) after each slide. This approach meant that correct answers had to be actively produced by the students. The written answers were evaluated manually and blindly by the first author. Correct answers were rewarded with one point. The test score was calculated as the sum of all the correct answers (max. 6 points). At the end of the study, students received sample solutions as feedback to reward them for their participation (see table 1 (Tab. 1)).

### Procedure for data analysis

Visualizations of the eye-tracking data were performed using RStudio software (Version 1.3.1093, RStudio Team, 2020) with the scan path extension [61]. Statistical analyses were performed with SPSS version 28 (IBM Corp., Armonk, NY). All statistics were performed under a significance value of α=0.05 and specified by a two-tailed *p value*, and an effect size (partial) η^2^. A η^2 ^greater than 0.14 was considered a strong effect. To capture mean differences, a two-sided t-test or ANOVA (>2 variables) for was performed with Bonferroni correction for multiple testing to counteract the likelihood of incorrectly rejecting a null hypothesis. To identify the discrete predictive value of each eye movement variable (independent variables) with the test score (dependent variable), a multivariate regression analysis was performed for each timepoint. 

## Results

### RQ1: After strict preprocessing, the webcam eye-tracking shows an acceptable data quality

**Figure 3:**
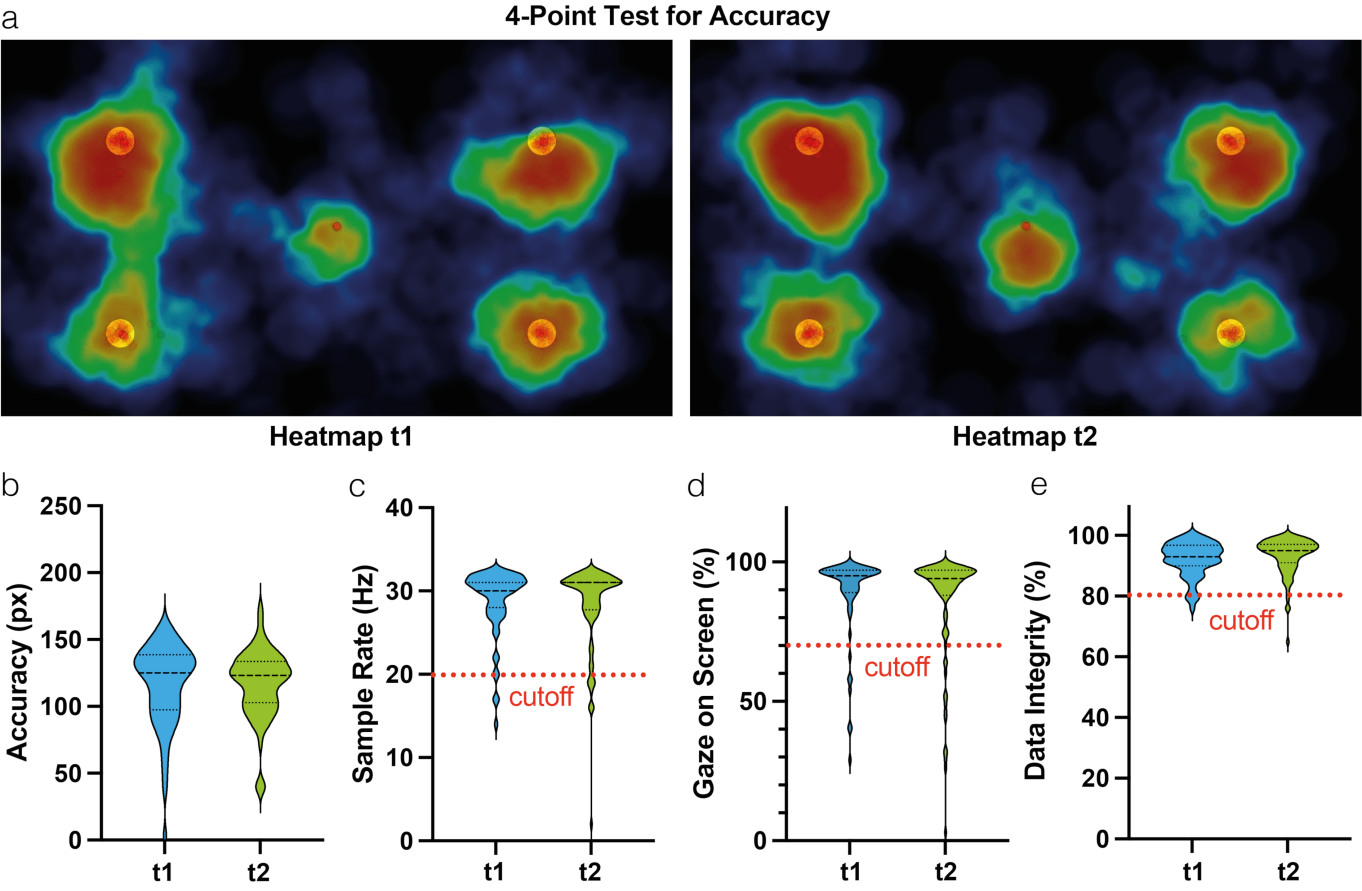
Webcam eye-tracking methodology – data quality. (a) Fixation-based heatmaps for testing accuracy for timepoints one and two. Data are aggregated per group. The color of the point cloud corresponds to the intensity of the subjects’ gaze (red=strong; blue=weak). (b) Accuracy is quantified and expressed in pixels. (c) Sample rate in Hz and *mean* gaze on screen time in % are shown as additional indicators for the data quality at t1 and t2, respectively. The red dotted line marks the cutoff for study inclusion. Participants with either a sample rate<20 Hz or a gaze on screen rate<70% were excluded in the preprocessing phase.

The 4-points test for accuracy showed gaze clouds on all four dots (yellow circles) (see figure 3 a (Fig. 3)). The gaze intensity is illustrated by different colors (red>yellow>green>blue>black), while the gaze cloud in the center of the screen corresponds to the central fixation bias. At both timepoints, there was a small off-set located downward in the upper quadrants. The click-to-gaze accuracy was suitable across both time points, with a *mean*=115.7 px±31.1 for t1 and *M*=116.9 px±25.8 for t2 (see figure 3 b (Fig. 3)). This value represents the deviation of the target point and the actual gaze position with a smaller value indicating a higher level of accuracy. A sampling rate of the participants’ webcams was in the range of 14-32 Hz (*M*=28.8 Hz±4.1) for t1, and 2-32 Hz (*M*=28.3 Hz±5.1) for t2 (see figure 3 c (Fig. 3)). The participants’ gaze-on-screen rate ranged from 29-99% (*M*=88.8%±15.3) for t1 and 3-99% (*M*=86.0%±19.5) for t2 (see figure 3 d (Fig. 3)). The data integrity (completeness of the data) at t1 was *M*=92.17%±5.98 and for t2 *M*=93.35% 6.01; thus, approximately 7-8% of the data were lost in both timepoints (see figure 3 e (Fig. 3)). Reasons for this loss may include detection difficulties and eye blinking.

### RQ2: Webcam eye-tracking measures visual expertise development in curricular histology training

**Figure 4:**
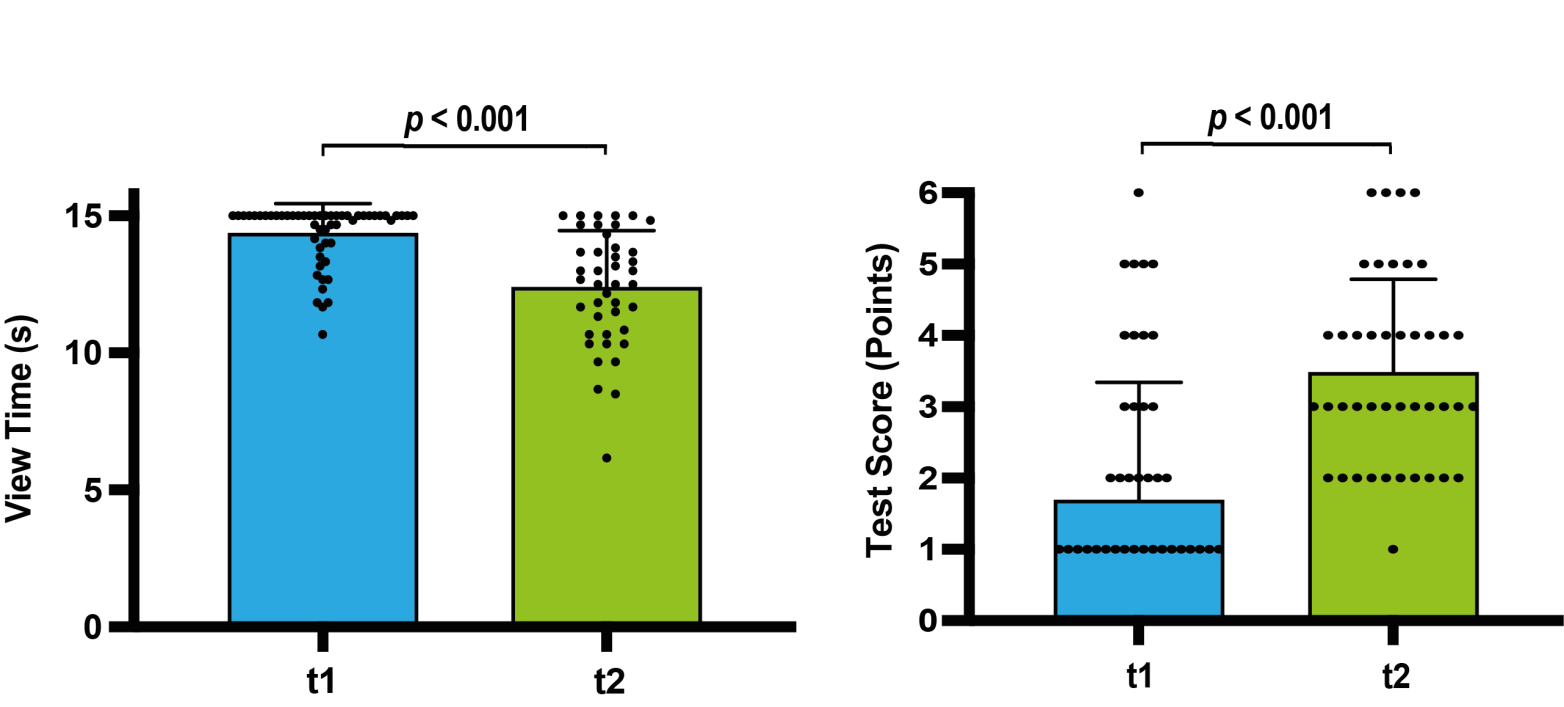
Online histology course – performance data. The results for timepoint 1 are shown in blue, and those for timepoint 2 are shown in green. The *P*-value shows the result of the statistical testing. Each circle represents one participant. Columns with the *mean* and *standard* *deviation* show the *mean* view time on the slides in seconds (maximum 15 seconds) and the mean test score in points (x out of six questions). *t1=timepoint 1 after 10 course sessions; t2=timepoint 2 after 20 course sessions*.

**Figure 5:**
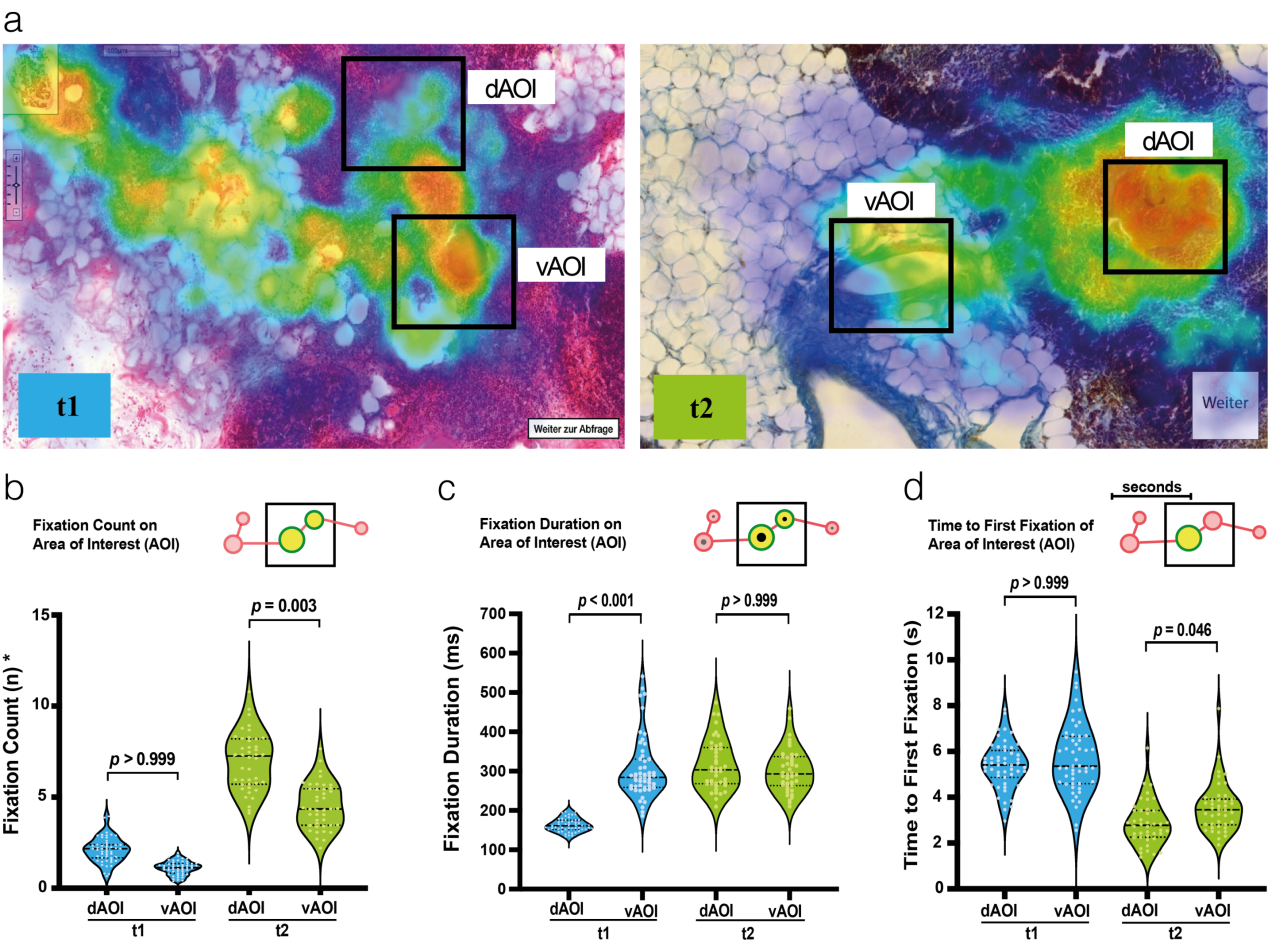
Webcam eye-tracking methodology – eye metrics. (a) An overlay of all participants’ fixations of the range 0.5-5 seconds of image viewing are shown as a fixation-based heatmap for both timepoints, respectively. (b-d) The results for t1 are shown in blue, and t2 are shown in green. The *P*-value shows the result of the statistical testing. Each circle represents one participant. The median and 25^th^-75^th^ percentile are shown for the violin plots. (b) Violin plots illustrate the fixation count on the dAOI and the vAOI for both timepoints, respectively. (c) Violin plots illustrate the fixation duration on dAOI and vAOI for both timepoints, respectively. (e) Violin plots illustrate the time to first fixation on dAOIs and vAOIs fixation. Abbreviations: *=View time adjusted values were used; dAOI=diagnostically most relevant areas of interest; vAOI=visually most salient but diagnostically irrelevant areas of interest; t1=timepoint 1 after 10 course sessions; t2=timepoint 2 after 20 *course sessions*.

The first analysis aimed to show whether the students improved in visual expertise over the course span (see figure 4 (Fig. 4)). The following analyses on eye-tracking metrics were conducted to show that the webcam eye-tracking can reflect this development (see figure 5 (Fig. 5)).

#### Testscore increases and view time decreases from t1 to t2

The test score increased (t(91)=5.69, *p*<0.001, η^2^=0.26) from* M*=1.69 points±.69 at t1 to *M*=3.48 points±1.31 at t2, which indicates an improvement in diagnostic accuracy in the slide identification task. The analysis of the total view time for t1 (*M*=14.35 s±1.09) and t2 (*M*=12.38 s±2.08) showed a decline (t(91)=5.99, *p*<0.001, η^2^=0.28) (see figure 4 (Fig. 4)). There was a ceiling effect for the view time and a floor effect for the test score at both timepoints (t1>t2), which suggest that the task was challenging for the students who were under high level of time-pressure.

#### The fixation count on dAOI is higher in t2

There was no difference between the fixation counts of the dAOI and those of the vAOI at t1 (F(51)=1.74, *p*>0.999), which suggests that students could not effectively distinguish between visually salient but irrelevant areas and diagnostically important areas (*mean* fixation count for dAOI=2.10±0.69 vs. vAOI=1.12±0.37) (see figure 5 b (Fig. 5)). Students at t2 showed an increased orientation toward the dAOI (F(42)=3.53, *p*=0.003, η^2^=0.43), thereby affirming information-reduction theory (mean fixation counts for dAOI=6.96±1.56 vs. vAOI=4.50±1.25). 

#### The fixation duration on dAOI is higher in t2

Students at t1 showed a lower fixation duration on the dAOI than on the vAOI (F(51)=14.26, *p*<0.001, η^2^=0.74), which indicates a lower interaction rate with the diagnostically relevant areas (see figure 5 c (Fig. 5)). However, in t2, the fixation duration on the dAOI increased so that the difference with the vAOI was no longer significant (F(42)=1.28, *p*>0.999); this indicated a higher detection rate of diagnostically relevant regions at t2. These results showed an increased ability of the trained students to interact with dAOIs; however, they were still occupied with vAOIs.

#### The time to first fixation of dAOI is lower in t2

At t1, there was no significant difference between the dAOI (M=5394 ms±1025) and the vAOI (*M*=5696 ms±1515) (F(51)=1.29, *p*>0.999), while at t2, the time to first fixation for the dAOI (*M*=2862 ms±965) was lower than that for the vAOI (*M*=3557 ms±1094) (F(51)=2.69, *p*=0.046, η^2^=0.10) (see figure 5 d (Fig. 5)). Together, students at t2 were able to detect dAOIs faster than vAOIs, which is in line with the holistic theory of visual expertise.

### RQ3: Eye movements predict test performance in the slide identification task

Eye movements recorded by the webcam eye-tracking could predict the test scores at t1 (see table 2 (Tab. 2)). In other words, the statistical model was able to predict the corresponding test scores of the students by their eye movements. Approximately 39% of the test score variance (R^2^_adj_=0.392, *p*<0.001) was explainable by nine eye-movement variables. At t2, the statistical test result merely misses significance (R^2^_adj_=0.103, *p*=0.057), which indicates the lower predictive power of the model. The predictors had similar regression coefficients at both second points. These results suggest that eye metrics were robust predictors for early visual expertise. Moreover, we observed that the predictive power is higher at an early timepoint than later in the course.

## Discussion

The aim of this study was to test the use of webcam eye-tracking in an (online) histology curriculum. 

### How accurately can webcam eye-tracking detect eye movements?

To overcome some of the existing limitations of laboratory eye-tracking settings (expensive hardware, artificial laboratory environment, small sample sizes), open-source webcam-based eye-tracking has been refined over recent years [44], [45], [50]. Our study supports the use of this methodology and demonstrates that the quality of data collected in a curricular online-only course is satisfactory. Setting up the test environment was convenient and did not require any programming skills. We were able to establish the research environments using the graphical interface, equivalent to a “drag-and-drop” principle. Care should be taken in future studies to ensure that the *areas of interest* are of sufficient size, i.e., large enough to compensate for potential accuracy errors. Due to the limited data quality of webcam eye trackers (see figure 3 (Fig. 3)), strict criteria must be applied to the data quality. We arrived at the comparatively high exclusion rate of approximately 30% of participants, which means that a high number of subjects must be recruited for such studies in order to obtain valid results. This is a reasonable expectation given the ease with which this methodology can be used in a classroom context. We believe that future improvements in webcam technology itself will further resolve the data quality issues. The students’ interest level in participating in the study was high, with even more students participating at the second timepoint compared to the first. Hence, implementation and use in (distant) classroom settings could become a practical possibility for medical educators.

### How valid is webcam eye-tracking in regard to capturing changes in visual expertise?

Our findings support the hypothesis that webcam eye-tracking can provide insights about temporal changes in visual expertise [4], [26], [37]. We could show that with the progress of the online histology course, students enrolled showed:

1. better test scores,
2. reduced slide view time,
3. more frequent fixations of diagnostically relevant areas,
4. longer fixation durations of diagnostically relevant areas, and
5. faster detection of relevant image areas.

Subsequent to *holistic processing*, the trained students focused on relevant areas indicating improved pattern recognition skills and expertise-related top-down control (see figure 5 d (Fig. 5)) [38], [51]. Furthermore, the students were more confident in distinguishing relevant from irrelevant areas, which can be interpreted as an improvement in visual expertise according to the *information reduction model* (see figure 5 b-c (Fig. 5)) [19]. The observable improvements occurred over the course span of ten course sessions, thereby highlighting the importance of early visual expertise development in histopathology training. To our knowledge, this study is the first to measure the development of visual expertise in histology training with a longitudinal study design. Given the paucity of literature, further research is needed to provide more insight on these important early stages.

### How reliably can webcam eye-tracking distinguish between low and high performances?

Combining several eye measurements, our linear models predicted up to 39% of the test score variance, which is an unexpectedly prediction level for such a complex cognitive task. A particularly interesting observation is that the predictive power of eye movements decreased with an increase in training duration (see table 2 (Tab. 2)). Therefore, the use of the webcam eye-tracking might be of particular value at the beginning of the training to monitor early visual expertise development. Future eye-tracking studies will investigate the presence of different search profiles in histology, at what point they develop, and how they affect students’ visual searching behavior and performance. Another source of methodological triangulation would be desirable to fully understand the various cognitive processes conducted during this development (e.g., qualitative think-aloud protocols) [40].

### Possible areas of implementation in medical education

Although there are still many open questions, it is worth discussing implementation options early on. Medical educators may use this methodology to gain insight into learners’ unconscious perceptual mechanisms in a range of professional settings, including histopathology, surgery, and radiology. For example, webcam eye-tracking technology could be used as a practical and cost-effective method for evaluating the effectiveness of curricula. This would allow subjective self-report data to be easily supplemented with objective performance data [57]. The affordable purchase price would enable the equipment to be installed throughout whole classes. Thus, it would be possible to use webcam eye-tracking across the board to provide real-time feedback to the instructor regarding task difficulty [7], cognitive load [43], [55], or students’ in-class attention [37]. This information could help to evaluate which educational methods may be problematic and at what point a change in method is appropriate. These data could also be evaluated post hoc, for example, to revise lecture slides and identify particularly difficult parts during a lecture. With sufficiently large samples, valuable feedback on the level of visual expertise could be given both to the students themselves and to the lecturers [20]. This method could be especially valuable in online learning environments, where feedback is more challenging due to technical limitations, as we experienced in the COVID-19 pandemic [9], [10]. 

This methodology opens up further opportunities for educational scientists. The ease of implementation enables to measure a larger number of subjects in a short time. Thus, eye-tracking studies can recruit more participants. This approach consequently allows large (online) studies to detect smaller effects that have previously gone undetected due to small sample sizes or to conduct longitudinal studies [16]. Another valuable source for methodological triangulation could be the use of scroll or zooming data during image inspection, which was successfully applied by van Montfort et al. [60] and den Boer et al. [11]. Finally, this methodology is open source, which allows countries and faculties with limited financial resources to benefit from it.

### Limitations

Along with these findings, several limitations should be acknowledged. It is currently undisputed that the webcam eye tracking method provides lower quality data than that provided by conventional laboratory eye trackers [50]. To anticipate this limitation, we recruited more participants and applied stringent procedures for data quality. Furthermore, as the study’s participation was voluntary, we cannot exclude our cohorts from selection bias. Even though we attentively inspected the time stamps, and screen resolutions for such conspicuity, we cannot rule out that certain students participated in the study multiple times. 

## Conclusion

This is the first study to examine the use of webcam eye trackers in an educational context, and on a larger sample of medical students pursuing an undergraduate histology training. The webcam eye-tracking suggested both accuracy in measuring visual expertise in histology, and value for the in-depth evaluation of (online) curricula. As technology continues to advance, the implementation of this methodology can be used to tap into previously unused potential that is likely to be leveraged in future variations of outcome-based course formats.

## Funding

This study did not receive any specific grant from funding agencies in the public, commercial, or not-for-profit sectors. Our work in the development of digital histology learning resources is funded by the Land Nordrhein-Westfalen under the grant “OERContent.NRW” (Projekt “Digital Histo NRW – Digitale Histologie in der Hochschulmedizin, Bio- und Gesundheitswissenschaften in NRW”). 

## Competing interests

The authors declare that they have no competing interests. 

## Supplementary Material

Supplemental material

## Figures and Tables

**Table 1:**
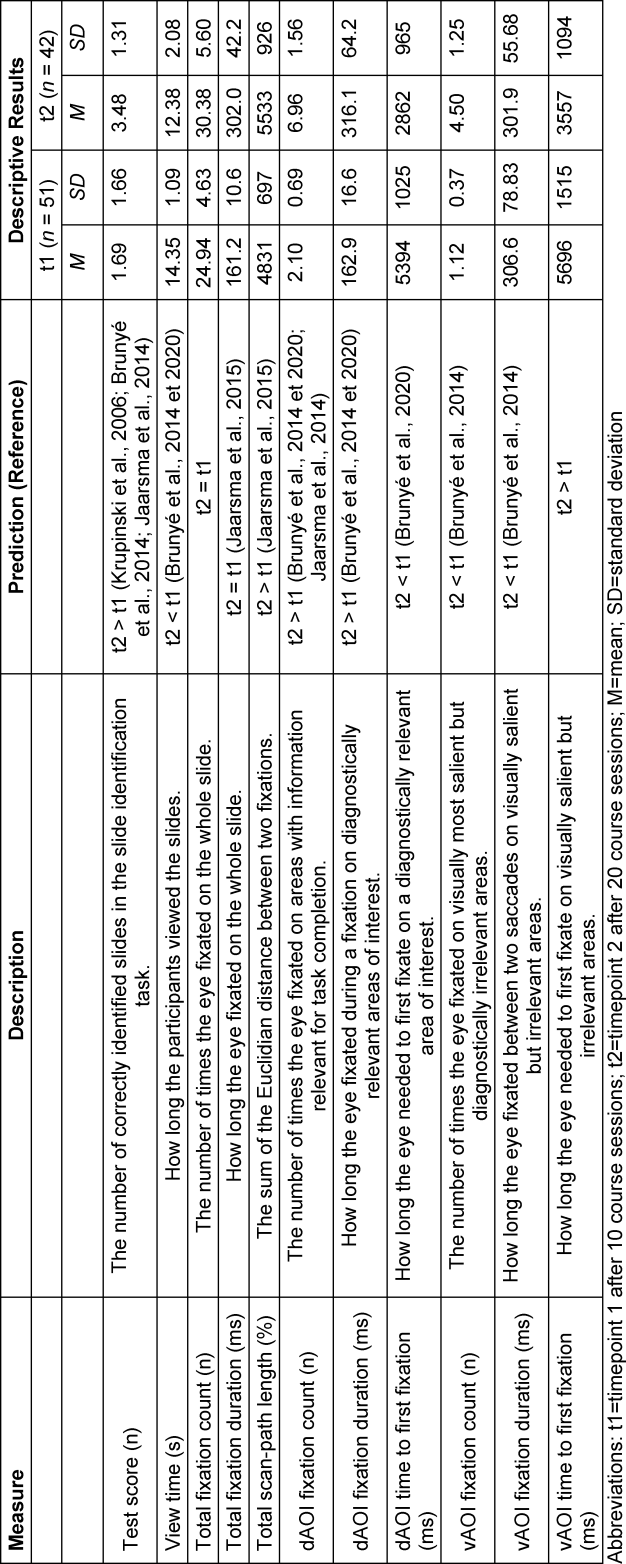
Variables used in this study, their description, respective units, prediction, and descriptive statistics

**Table 2:**
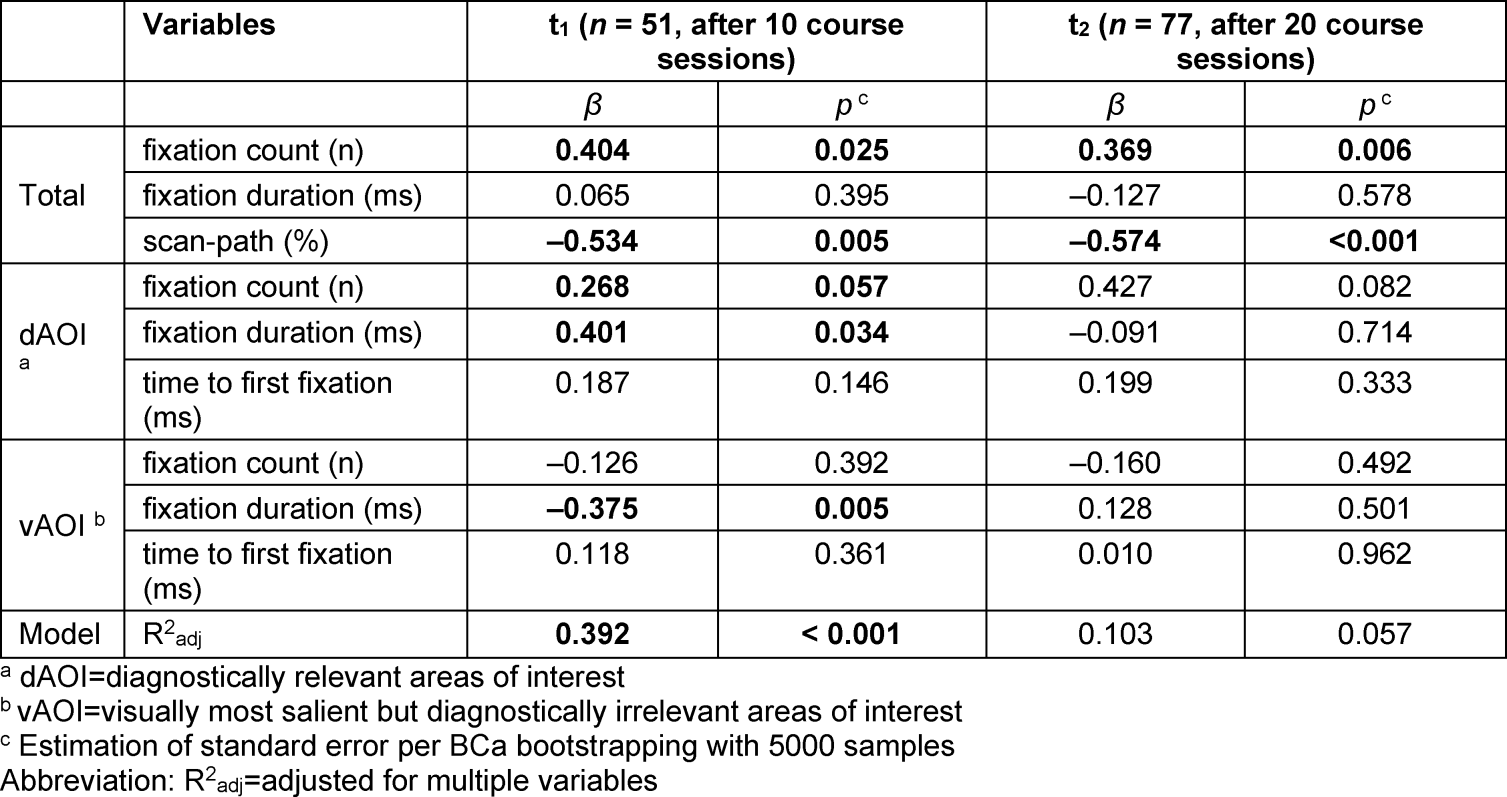
Predictive value of eye metrics on the test scores at timepoints t1 and t2. Deviation of β from zero is associated with a higher test score in the slide identification task
