# Supplementary material for: Webcam-based eye-tracking to measure visual expertise of medical students during online histology training
Source: GMS J Med Educ. 2023 Sep 15;40(5):Doc60. doi: 10.3205/zma001642 (PMC10594038; doi:10.3205/zma001642)
Supplement: Supplemental material [file JME-40-60-s-001.pdf]

Attachment 1: Supplemental material

Evidence before this work

To date, there are already a few promising studies that have investigated the technical implementation for webcam eye-tracking methodology and demonstrated at least satisfactory data quality. Semmelmann and Weigelt [50] for instance, showed suitable data quality during fixation, pursuit, and free-view tasks. Bott et al. [3] reported high correlations of r=.81 when using webcam eye trackers and laboratory eye trackers at the same time. Bánki et al. [2] compared webcam with laboratory eye-tracking in capturing infants' viewing behavior and reported positive results after careful quality control. Although these initial studies are encouraging, there is no research on the use of such technology in an educational context or on potential areas of implementation in medical education. In this context several questions arise. For example, are students willing to use webcam eye-tracking methodology and follow the instructions properly at home? As students use different hardware (laptops and webcams), and conduct these studies in unsupervised environments, how high is the data quality? If the quality is suitable, can the results be used to describe the development of visual expertise among medical students ("within-subject")? How well can the results differentiate between several learners ("between-subject")?

Power analysis to determine the sample size

Assuming a medium effect size of d=0.5, an α=0.05, and a power ($1-\beta$) of 0.80, the sample size estimate suggests a minimum of N=34 participants per group for t tests. For multiple linear regression assuming a medium effect size of f^2=0.15, an α=0.05, and a power ($1-\beta$) of 0.80, N=68 participants were needed.

Details on participating students

From the approximately 180 students enrolled in the course, 74 participants (=100%) endured the calibration phase properly at t1, and 63 participants completed the test and provided results (=85%). At t2, 115 participants (=100%) passed the calibration phase, and N=95 provided results (=83%). Altogether, N=42 students (age mean 21.49±1.92 years; 28 females) participated in both measurements at t1 and t2. Participation in this study was voluntary, anonymous, and had no impact on the students' grades.

Description of the online-only histology curriculum

Due to physical-distance restrictions during the COVID-19 pandemic, the entire curricular course was taught in a synchronous online format. In total, there were 20 course days, with three hours of instruction per course day. In addition to the content on cell biology and basic tissue types (course days 1-4), almost all organs were systematically addressed in the subsequent course days (7-20). The curricular histology course was held online only using videoconferencing tools and virtual microscopy, as previously described [10]. Students had off-campus access to virtual microscopy (an open microscopy environment) that contained all the relevant course slides [22]. As a result, we anticipated a higher level of engagement with the course slides and a significant increase in visual expertise by the end of the course.

Measures to ensure high data quality

Since the quality of the data plays a central role in the webcam eye-tracking methodology, special attention was given to increasing the quality [25]. A run-through pilot study was performed with two novices to optimize the eye-tracking performance. Here, the main focus was to adjust the duration of the presentation time and to assess the behavior of the participants during the study. The participants were encouraged to pay attention to good lighting and maintaining a steady head position. If the participant’s head slid aside, the study was automatically paused, and the participants were encouraged to reposition. The students were asked to sit up straight and maintain a constant distance from the webcam, to provide enough light, to keep their heads still and to avoid possible distractions.

Webcam eye-tracking technology assumes that the position of the eyes corresponds to the position of the mouse click [44], [45]. A 40-point calibration was performed proportionally at the beginning of the study using 40 clicks and cursor movements with three different background colors (13 points each with RGB #FFFFFF, #000000, #919191), followed by a 4-point test for accuracy. To pass the accuracy test, the participant's gaze predictions for each point had to be greater than 50% within a 200 px tolerance radius of that point. The trial was terminated if calibration was not successful after five attempts. Due to the rapid nature of the study (around 10 minutes), no recalibration was performed and no rest breaks were taken. In between the two slides, a separator was used that showed a crosshair in the middle of the screen to relax the retina and fixate the eye positions to the center. The interstimulus interval was 1.5 s. Sufficient eye-tracking data quality was obtained considering accuracy, sampling rate, and data integrity, and gaze-on-screen rate.

Accuracy was defined as the average difference between the mouse click and the measured gaze position measured in pixels (related to the concept of internal validity). Precision was defined as the dispersion measure of standard deviation (related to the concept of reliability). The sampling rate reflects the number of gaze position measurements made per second in Hz. Data integrity refers to the completeness of the data as the percentage of the sample that provides coordinates for the gaze signal. Missing data were linearly interpolated, and gaze positions were denoised with a reduction level of 21 (=median was calculated for 21 consecutive points). Visual angles could not be computed, as no accurate estimates were available for the participants' distance to the screen. No chinrest was used. Gaze position data were measured in pixels. Most students used a screen resolution of 1440x900 px (t_1=22%; t_2=36%), 1536x864 px (t_1=11%; t_2=16%), or 1280x720 px (t_1=9%; t_2=16%). As the resolutions of participants' screens varied, the gaze positions were normalized to height and width (in %). The first 0.5 seconds of the data were omitted for statistical testing to avoid central screen bias. Fixation events were detected using an algorithm similar to the I-VT fixation filter (using angular expressed as a percentage of the item size instead of angular velocity) [41]. Algorithm-detected events were manually double-checked. The minimum fixation duration was defined as 100 ms [24].

Details of the stimuli

From a pool of histological slides (=stimuli), we selected those that had already been discussed previously in the course and that were particularly discriminative based on our experience. Thus, the pool consisted of approximately 60 different slides at timepoint 1 and 200 (60+140) slides at timepoint 2. A high number of slides may increase the difficulty of the slide identification task due to more potential differential diagnoses. The difficulty for the students at timepoint 2 was thus higher, since a larger selection was theoretically available. This in turn may cause an underestimation of the differences found in this study.

Details of the data analysis

To check for multicollinearity among the eye-tracking predictors, the variance inflation factors (VIF) were determined, which showed no problems with collinearity (VIFs<6). No prior transformation of the variables was performed. Instead, bootstrap analyses with BCa correction, and n=5000 samples were conducted to anticipate for nonnormality distributions [53]. For unknown distributions, bootstrapping may be used as a resampling approach that generates multiple simulated samples to estimate the sampling distributions.

Description of the eye-tracking variables

The eye measurements of fixation count, fixation duration, and scan-path length (the sum of the length between two fixations in % of the screen) were included to measure in-depth processing (see table 1). To ensure comparability, fixation counts from students who finished the slide identification task earlier were linearly extrapolated (=*view time–adjusted values*) for the analyses related to research question 2. This accounts for differences in view time, and allows a fair comparison. For the regression models needed to address research question 3, time-unadjusted values were used to partialize out the view time. Capturing *saccades* (rapid eye movement from one point to another) can usually be helpful in capturing holistic processing [51]. However, due to low *mean* sample rates under 30 Hz (see figure 3), we abstained from detecting saccades or other fast events [13].

Defining areas of interest

Considering the comparable difficulty and sample preparation staining at both time-points, the slides showed regions at different magnification depths (see figure 2). Areas of interest were used to link eye-movement measures to certain parts of the image. Slides and dAOIs were previously selected by expert consensus (four histology teachers with > 15 years of experience in the field of histology teaching), and only those slide regions that contained approximately 1-3 distinct areas of interest were chosen. The vAOIs were chosen using automatically generated saliency maps (OpenCV Saliency Detection) (Rosebrock, 2018) and were manually double-checked. All the AOIs across the images had comparable sizes (between 15-20% of the slide), and the dAOIs and vAOIs on the same slide were identical in size (see figure 5a). Overall, the dAOIs were larger than the potential stimulus object to compensate for inaccuracies. We did not use Masson Goldner staining to consider red-green

color gradients for students with color blindness, and we chose slides previously taught in the course. The identification of the slides was unique, and the dAOI and the vAOI did not overlap. Fixations were considered part of an AOI if the fixation started in the AOI range. Webcam eye-tracking shows the lowest accuracy at the bottom corners of the screen. Additionally, the central point of the screen is artificially fixated at the beginning of the presentation (central bias) [13]. To counteract both potential confounders, the AOIs were placed between the center and the corner of the screen (see figure 5a).

Supplementary Table 1: Item analyses for the stimuli in the slide identification task

Timepoints	Item difficulty	*95% CI*	*SD*	Corrected Item-Total Correlation	Cronbach's alpha
t1 (*n* = 6)					0.66
slide 1	0.33	0.21;0.46	0.48	0.34	
slide 2	0.23	0.12;0.34	0.43	0.34	
slide 3	0.17	0.07;0.26	0.38	0.51	
slide 4	0.15	0.06;0.24	0.36	0.54	
slide 5	0.30	0.18;0.42	0.46	0.31	
slide 6	0.35	0.23;0.47	0.48	0.40	
t2 (*n* = 6)					0.47
slide 1	0.82	0.74;0.89	0.39	0.28	
slide 2	0.62	0.21;0.40	0.49	0.16	
slide 3	0.31	0.21;0.40	0.46	0.20	
slide 4	0.20	0.12;0.28	0.40	0.34	
slide 5	0.84	0.84;0.77	0.37	0.17	
slide 6	0.47	0.37;0.57	0.50	0.27	

Abbreviation: M = mean; CI = confidence interval; SD = standard deviation

Supplementary Figure 1

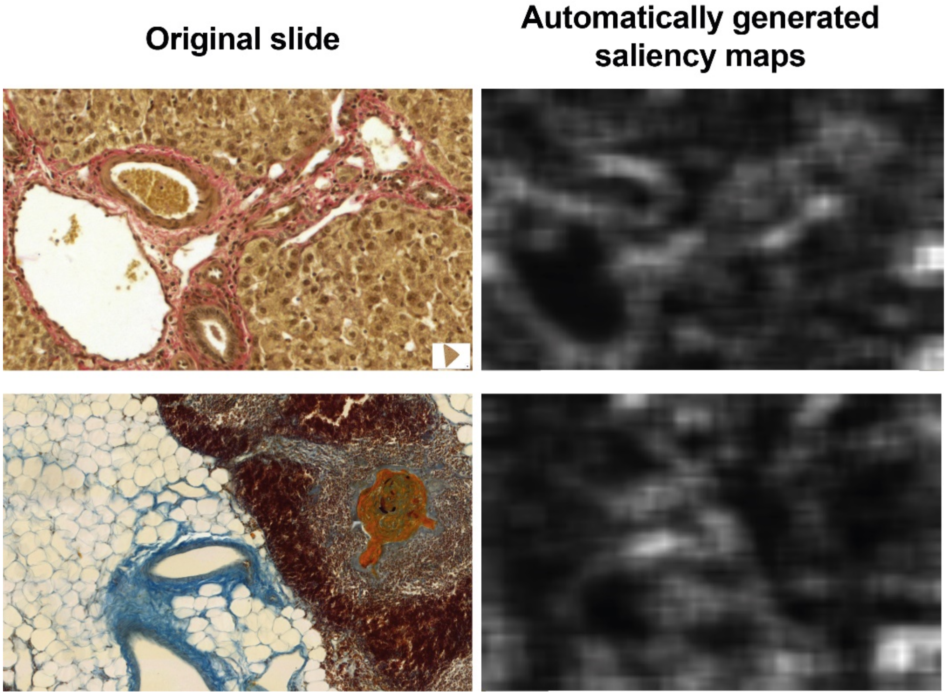

Attachment 1 to Darici D, Reissner C, Missler M. *Webcam-based eye-tracking to measure visual expertise of medical students during online histology training*. GMS J Med Educ. 2023;40(5):Doc60.
DOI: 10.3205/zma001642
